# Incubation of semen with human follicular fluid improves the antioxidant status and quality of spermatozoa after freezing–thawing

**DOI:** 10.1530/RAF-24-0056

**Published:** 2025-06-26

**Authors:** Monireh Mahmoodi, Elham Shojafar, Maryam Dastjani-Farahani

**Affiliations:** Department of Biology, Faculty of Science, Arak University, Arak 3848177584, Iran

**Keywords:** human follicular fluid, human sperm freezing and thawing, oxidative stress, antioxidant

## Abstract

**Graphical abstract:**

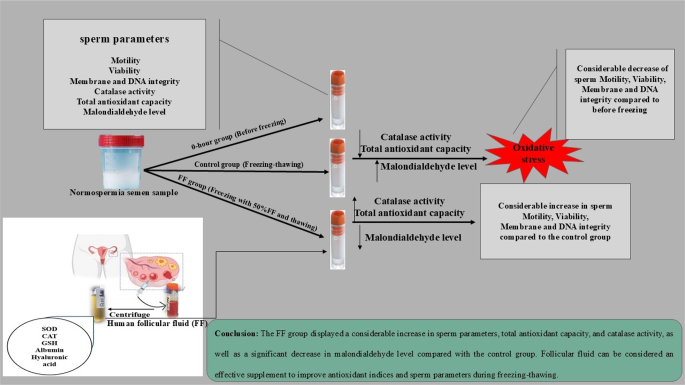

**Abstract:**

The sperm freezing–thawing procedure is the most commonly used technique in clinics to preserve male fertility before any pathological destruction of the testis. Therefore, most studies are currently focused on optimizing this method to achieve high-quality semen after thawing. During cryopreservation, oxidative stress-induced damage affects sperm structures and decreases their fertility potential. The use of antioxidants in freezing media can protect sperm against oxidative damage. We designed this study to evaluate whether incubation of semen with human follicular fluid, which contains a wide variety of enzymatic and nonenzymatic antioxidants, can prevent the negative effects of freezing–thawing on human spermatozoa. Human semen was divided into three groups i) the 0-hour group (before freezing), ii) the control group (after freezing–thawing), and iii) the FF group (after freezing with 50% follicular fluid). The sperm motility, viability, integrity of the plasma membrane and DNA, mitochondrial membrane potential, malondialdehyde level, total antioxidant capacity, and catalase activity were assessed in these three groups. The findings showed a significant decrease in sperm motility, viability, plasma membrane and DNA integrity, mitochondrial membrane potential, total antioxidant capacity, and catalase activity and a significant increase in malondialdehyde level in the control group compared with the 0-hour group. The FF group displayed a considerable increase in sperm parameters, total antioxidant capacity, and catalase activity and a significant decrease in malondialdehyde level compared with the control group. Follicular fluid can be considered an effective supplement to improve antioxidant indices and sperm parameters during freezing–thawing.

**Lay summary:**

Sperm freezing is a useful method in clinics to preserve fertility in people who are affected by some problems such as diseases or chemotherapy which decrease their fertility. Although various studies are focused on optimizing this method, some challenges decrease the efficiency of this method. Oxidative stress has been reported as one of the mechanisms inducing negative effects on sperm during freezing–thawing. Therefore, the use of cryoprotectants and also some antioxidants has been suggested to increase sperm quality during freezing–thawing. In this study, we used human follicular fluid before freezing to assess sperm parameters. Our results showed that follicular fluid with antioxidant properties and other proper factors can have positive effects on human sperm during freezing–thawing and could be proposed to be added to the sperm freezing medium to improve sperm parameters, although this suggestion needs to be confirmed by further experiments.

## Introduction

Sperm cryopreservation, as an important procedure, is widely used in assisted reproductive technology (ART) to preserve male fertility before chemotherapy, radiotherapy, or any pathological conditions that impair the function of the male reproductive system and sperm (Riva *et al.* 2018). Despite the many advantages of the freezing and thawing method in ART, studies have reported that the process of cryopreservation causes various structural, biochemical, and functional damage to spermatozoa, which could affect the fertility potential of thawed sperm (Todorovic *et al.* 2022). It is illustrated that sperm cryopreservation not only induces adverse effects on motility and viability, plasma membrane lipid composition, and acrosome integrity, but also leads to sperm DNA fragmentation and cell death (Mandal *et al.* 2014, Rarani *et al.* 2019). Oxidative stress induced by the excessive production of reactive oxygen species (ROS) is known as a possible mechanism of sperm damage during cryopreservation (Gualtieri *et al.* 2021). In fact, in this method, an imbalance in water movement changes cell volume and induces osmotic stress (Ball 2008). In addition, cold shock, removal of seminal plasma, and the formation of intracellular ice crystals are also attributed to ROS generation and the induction of oxidative stress (Pérez-Pé *et al.* 2001).

Spermatozoa are particularly vulnerable to oxidative stress due to the specific structure of the plasma membrane and mitochondria and the low amount of cytoplasm and antioxidant factors (Sabeti *et al.* 2016). Given that, after thawing, the quality of sperm can be associated with a higher rate of pregnancy in ART methods; it is important to preserve sperm fertilizing ability during the freezing and thawing procedure. Currently, researchers widely use antioxidants to remove free radicals and oxidative stress factors to optimize the freezing and thawing method and improve sperm quality after thawing.

The application of cryoprotectants and antioxidants before cryopreservation or in the freezing medium is known as a main protection strategy to decrease oxidative stress-induced damage and improve sperm cryosurvival (Mehdipour *et al.* 2022). Studies have proven that antioxidants such as vitamin E, leptin, vitamin C, and melatonin in the freezing medium can protect sperm against cryoinjury and enhance sperm quality (Kalthur *et al.* 2011, Karimfar *et al.* 2015, Fontoura *et al.* 2017, Moreira *et al.* 2022).

Follicular fluid (FF), a biological fluid obtained from women after controlled ovarian stimulation, is secreted from the granulosa cells, thecal cells, and blood surrounding the ovary (Wang *et al.* 2022) to provide a valuable microenvironment for oocyte maturation, follicle development, implantation, early embryo development, and germ cell–somatic cell communication (Yang *et al.* 2022). FF consists of steroids, metabolites, prostaglandins, polysaccharides, lipids, proteins, small peptides, growth factors, and ROS, which reflect the metabolic state of follicles (Bender *et al.* 2010). In addition, FF contains several antioxidants that play crucial roles in protecting oocytes against oxidative stress-induced harmful effects (Ambekar *et al.* 2013, Kushnir *et al.* 2016). The presence of catalase (CAT), superoxide dismutase (SOD), glutathione S-transferase (GST), peroxiredoxins (Prx), and glutathione reductase has been detected in FF samples (Angelucci *et al.* 2006, Twigt *et al.* 2012, Ambekar *et al.* 2013). Moreover, glutathione (GSH), vitamin E, and albumin, and the active enzymatic and nonenzymatic antioxidants, have also been detected in FF (Da Broi *et al.* 2016, Freitas *et al.* 2017). Follicular fluid also contains various biological substances, including hyaluronic acid (Babayev *et al.* 2021), anti-sperm antibodies or immunoglobulins (Saragüeta *et al.* 1994), progesterone (Kreiner *et al.* 1987), and platelet-activating factor (Amiel *et al.* 1991), which can affect the sperm parameters. In this context, previous studies have confirmed the positive effects of FF on sperm motility and hyperactivation (Getpook & Wirotkarun 2007), *in vitro* sperm chemoattraction (Wang *et al.* 2001), the sperm acrosome reaction and capacitation (Bravo & Valdivia 2018), and the integrity of sperm chromatin and DNA (Dorado-Silva *et al.* 2020).

Sperm motility, viability, and plasma membrane integrity are important parameters that can play a decisive role in ART and conception (Sati & Huszar 2015). In addition, sperm DNA fragmentation decreases pregnancy rates and can lead to impaired fetal development or affect offspring (Sedó *et al.* 2017); therefore, improving these parameters can increase sperm fertility and pregnancy. Previous studies have shown the positive effects of FF on sperm parameters *in vitro* (Bedaiwy *et al.* 2012), but the effects of FF on spermatozoa during cryopreservation are unknown. Given these facts, this study was conducted to investigate whether the addition of human FF, an antioxidant supplement, to semen before cryopreservation can protect it from cryodamage during freezing and improve vital sperm parameters after thawing.

## Materials and methods

### Preparing samples and grouping

According to the WHO guidelines (Gottardo & Kliesch 2011), human semen samples were obtained from 30 men with normospermia (25–45 years old) referred to the Academic Center for Education, Culture, and Research (ACECR), Qom, Iran. Consent was obtained from each patient after an explanation of the purpose and nature of all procedures used. This study was approved by the local ethics committee at the Arak University of Medical Sciences, Arak, Iran (IR.ARAKMU.REC.1400.248). After semen liquefaction, each semen sample was separated into three identical aliquots, which were randomly divided into three groups: i) the 0-hour group (sample was evaluated before freezing); ii) the control group (sample was evaluated after freezing and thawing (0.5 mL of semen + 0.5 mL of Ham’s F10 (Bioidea, Iran))); and iii) the FF group (sample was frozen with 50% FF and evaluated after thawing (0.5 mL of semen + 0.5 mL of FF)) (Yao *et al.* 2000). After 2 weeks, the motility, viability, plasma membrane and DNA integrity, mitochondrial membrane potential, lipid peroxidation, total antioxidant capacity, and catalase activity of the samples were examined.

### Follicular fluid collection and processing

Follicular fluids were obtained from 30 patients (25–45 years) undergoing the same IVF treatment (stimulated with a standard long protocol) at Rastak Fertility Clinic, Sina Hospital, Arak, Iran, after providing informed consent. In this study, follicular fluid was collected from individuals without infertility issues, specifically those undergoing fertility treatment due to male factor infertility. We ensured that all participants providing follicular fluid were within a similar age range and had undergone ovarian stimulation using the same protocol. Furthermore, sample collection procedures were standardized across all participants. These measures were implemented to minimize variability and ensure consistent conditions across all follicular fluid samples. The follicular fluid collection was performed during the oocyte retrieval procedure, and follicular fluid samples from individual follicles were pooled. After the procedure, the samples were centrifuged at 3,000 ***g*** for 10 min to remove debris and granulosa cells. Then, the follicular fluid supernatant was stored at −20°C for further experiments (Chen *et al.* 2016, Kurdi *et al.* 2023).

Cryopreservation was performed using a sperm freeze solution (Vitrolife, Sweden). Based on the protocol, 1 mL sperm freezing solution was slowly added to 1 mL each semen sample. The mixture was transferred to a cryovial and placed horizontally in nitrogen vapor for 15 min, quickly immersed in liquid nitrogen, and stored at −196°C for 2 weeks. After 2 weeks, the cryotubes were removed from the nitrogen tank and placed at 37°C, after which the samples completely melted. After thawing, the samples were washed with sperm washing solution (Vita, Ide Varzan Farda, Iran) at 300 ***g*** for 5 min and were used for further experiments.

### Sperm motility

Sperm motility was carried out according to the WHO guidelines (Gottardo & Kliesch 2011). A 10 μL sperm suspension was placed on a prewarmed slide, and sperm motility (progressive motility, nonprogressive motility, and none-motile sperm) was checked using a light microscope at 400× magnification. At least 200 sperm were counted in five fields, and total motility (progressive + nonprogressive motility) was counted, and the results were reported as a percentage.

### Sperm viability

According to the WHO guidelines (Gottardo & Kliesch 2011), one volume of sperm suspension and two volumes of 1% eosin (Merck, Germany) were mixed in the microtube and incubated at 37°C for 30 s. Then, an equal volume of 10% nigrosin (Merck, Germany) was added to the mixture, and a smear was prepared on a glass slide. After drying, the slides were observed using a light microscope at 1,000× magnification. 200 sperm were counted, and the percentage of viable sperm was calculated. In this method, live sperm heads are seen in white and dead sperm are seen in red.

### Sperm plasma membrane integrity

The hypoosmotic swelling test (HOS) was used to evaluate sperm plasma membrane integrity (Bahrami *et al.* 2020). 1 mL hypoosmotic solution (0.735 g sodium citrate dihydrate (Na_3_C_6_H_5_O_7_·2H_2_O; Sigma, USA) and 1.351 g fructose (Sigma, USA) in 100 mL distilled water) was placed at 37°C for 5 min, and 100 μL sperm suspension was then added to the solution. The mixture was incubated for 30 min, and a thin smear was then prepared on a glass slide. At least 200 sperm were evaluated, and the percentage of plasma membrane integrity was reported. In this method, sperm with a twisted tail had an intact plasma membrane. However, sperm with a straight tail were considered cells with a damaged plasma membrane.

### Sperm mitochondrial membrane potential (MMP)

The mitochondrial membrane potential was evaluated using rhodamine 123 (Rho) staining (Johnson *et al.* 1981). In this method, 5 μL rhodamine-123 (Sigma, USA) was added to 10 μL sperm sample and kept in a dark room at 25°C for 10 min. Then, the solution was washed with 1 mL phosphate-buffered saline (PBS, Sigma; USA) at 2,000 ***g*** for 10 min and the pellet was dissolved in 1 mL PBS. 10 μL suspension was smeared on a glass slide and 200 sperm were counted using a fluorescence microscope (Olympus, Japan) at 1,000× magnification. In this staining, the neck of sperm with healthy mitochondrial membrane potential is bright green and the neck of sperm with unhealthy mitochondrial membrane potential is colorless.

### Sperm DNA fragmentation

An SDFA kit (Ide Varzan Farda Company, Iran) was used to assess sperm DNA integrity. According to the kit protocol, the microtube containing the agarose gel was heated at 95–100°C. Then, 50 μL sperm suspension was added to melted agarose and mixed slowly. Then, 30 μL mixture was placed on a slide coated with agarose and the slide was kept at 4°C for 5 min. The slides were incubated with solution A and lysis solution for 7 and 15 min, respectively. The slides were washed with distilled water for 5 min and dehydrated with 70, 90, and 100% ethanol. The slides were stained with C solution for 2 min, D solution for 3 min, and E solution for 2 min. After washing, the slides were checked using a light microscope at 1,000× magnification and 200 sperm were counted. In this method, spermatozoa with intact DNA had large and medium halos, whereas spermatozoa with fragmented DNA had small or no halos.

### Biochemical evaluation of sperm

First, 20 μL sample was mixed with 50 μL NTPC lysis buffer (113 mM NaCl, 5.2 mM NaH_2_PO_4_, 5.2 mM Na_2_HPO_4_, 5.1 mM D-glucose, 1.7 mM CaCl_2_, 20 mM Tris and 4 mM EDTA solution in HCl, pH = 4.7, Sigma; USA) and centrifuged at 500 ***g*** at 4°C for 10 min. After washing, Triton X-100 (0.1%, Sigma, USA) was added to the sample and centrifuged at 7,000 ***g*** for 30 min (Hadwan 2008). Finally, the obtained supernatant was used to evaluate lipid peroxidation (MDA test), total antioxidant capacity (TAC), and catalase (CAT) activity.

#### Lipid peroxidation (MDA assay)

After the samples were prepared according to the Malondialdehyde (MDA) – TBARS Assay Kit (Colorimetric) (ZellBio, Germany), their absorption (OD) was read by an ELISA reader (Dana 3200) at 550 nm. Then, the MDA concentration of the sperm samples in different groups was calculated using a standard curve obtained from the absorption of standard solutions at different concentrations and reported as μmol/mL sperm.

#### Total antioxidant capacity (TAC)

Sperm total antioxidant capacity was assayed using the Total Antioxidant Capacity (FRAP) Assay Kit (Colorimetric) (ZellBio, Germany). After the preparation of samples according to the kit protocol, the samples were prepared, and the absorption (OD) of the samples was read by an ELISA reader at 593 nm. Then, the TAC of the sperm samples in different groups was calculated using a standard curve drawing by standard solution absorption and reported as mM.

#### Catalase (CAT) activity

Catalase activity was measured using the Catalase (CAT) Activity Assay Kit (ZellBio, Germany). According to the kit protocol, the absorption of the prepared samples was read by an ELISA reader at 550 ± 20 nm. By drawing a standard curve using the absorption of different concentrations of the standard solution, the activity of CAT was calculated according to the following formula:

Catalase activity (U/mL) = (ODblank - ODsample) × 371 × (1/60 × sample volume)

{ODblank: 0.03, sample volume: 20}

### Statistical analysis

The data were statistically analyzed using the SPSS software version 24 (Chicago, IL, USA). The means were compared by the repeated-measures analysis, and *P* ≤ 0.05 was considered statistically significant. The data are presented as the means ± standard error of the means (SEMs).

## Results

### Motility and viability of spermatozoa

**Figure 1:**
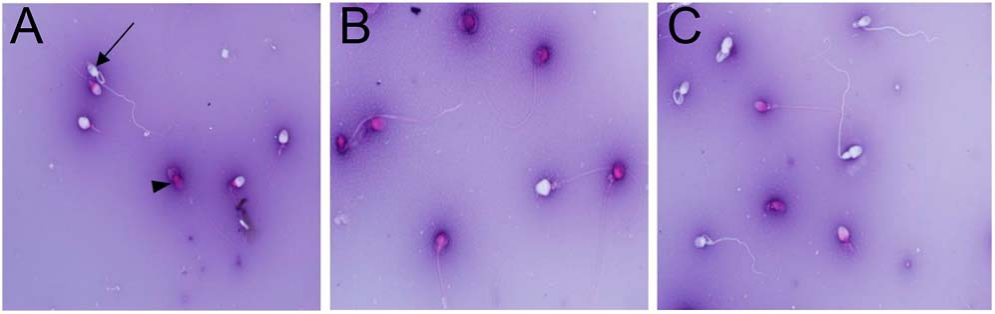
Evaluation of human sperm viability using eosin-nigrosin staining. A: 0-hour group, B: control group, C: FF group. Arrow: live sperm, arrowhead: dead sperm. Magnification: 1,000×.

Sperm motility and viability were significantly reduced (*P* ≤ 0.001) in the sperm freezing groups (control and FF) compared with the 0-hour group (before freezing) (Fig. 1 and Table 1). In the group incubated with FF (50%), sperm motility and viability were significantly (*P* ≤ 0.001) higher than the control group.

**Table 1:** Evaluation of sperm motility and viability in different groups of human spermatozoa. The data are presented as the mean ± SEM.

Groups*	Sperm motility (%)	Sperm viability (%)
0-hour	65.1 ± 1.44^a^	71.54 ± 1.89^a^
Control	29.1 ± 1.44^b^	40.17 ± 1.48^b^
FF (50%)	41.00 ± 1.42^c^	52.33 ± 1.52^c^

* 0-hour**:** sperm were evaluated before freezing; Control: sperm were evaluated after freezing; FF (50%)**:** sperm were frozen with FF (50%) and thawed after 2 weeks and evaluated.

Different subscripts (a, b, c) denote significant differences between groups (repeated measure analysis, *n* = 30 for each group, *P* < 0.05).

### Sperm plasma membrane integrity (HOSt)

Sperm plasma membrane integrity significantly decreased (*P* ≤ 0.001) in the control and FF groups compared with the 0-hour group. However, in the group incubated with FF (50%), sperm plasma membrane integrity was significantly (*P* ≤ 0.001) higher than the control group (Table 2).

**Table 2:** Evaluation of sperm plasma membrane integrity (HOS test), mitochondria membrane potential (MMP) (rhodamine-123 staining), and DNA fragmentation (SCD test) in different groups of human spermatozoa. The data are presented as means ± SEMs.

Groups*	Plasma membrane integrity (%)	MMP (%)	DNA fragmentation (%)
0-hour	68.12 ± 1.86^a^	73.45 ± 2.1^a^	15.48 ± 1.98^a^
Control	42.27 ± 1.45^b^	38.53 ± 1.70^b^	41.50 ± 1.53^b^
FF (50%)	51.47 ± 1.46^c^	49.63 ± 1.78^c^	28.17 ± 1.75^c^

* 0-hour**:** sperm were evaluated before freezing; Control**:** sperm were evaluated after freezing; FF (50%)**:** sperm were frozen with FF (50%) and thawed after 2 weeks and evaluated.

Different subscripts (a, b, c) denote significant differences between groups (repeated measure analysis, *n* = 30 for each group, *P* < 0.05).

### Sperm mitochondria membrane potential (MMP)

**Figure 2:**
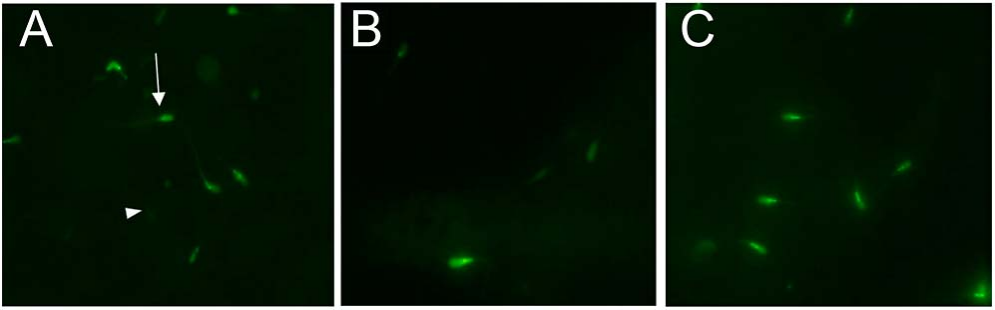
Evaluation of the human sperm mitochondrial membrane potential (MMP) using rhodamine-123 staining. A: 0-hour group, B: control group, C: FF group. Arrow: intact MMP, arrowhead: damaged MMP. Magnification: 1,000×.

The sperm MMP decreased significantly (*P* ≤ 0.001) in the control and FF groups compared with the 0-hour group. In the group incubated with FF (50%), MMP was significantly (*P* ≤ 0.001) higher than the control group (Fig. 2 and Table 2).

### Sperm DNA fragmentation

**Figure 3:**
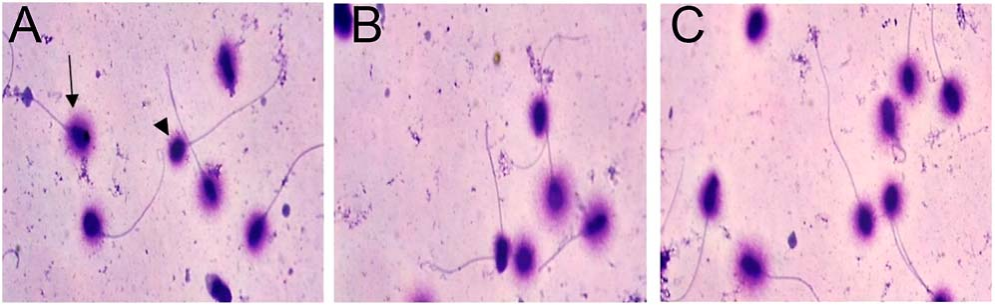
Evaluation of human sperm DNA fragmentation using the SCD test. A: 0-hour group, B: control group, C: FF group. Arrow: sperm with intact DNA, arrowhead: sperm with fragmented DNA. Magnification: 1,000×.

The percentage of sperm with fragmented DNA increased significantly in the control and FF groups compared with 0-hour group (*P* ≤ 0.001). However, DNA fragmentation was significantly (*P* ≤ 0.001) lower in the presence of FF than in the control group (Fig. 3 and Table 2).

### Lipid peroxidation (MDA) in sperm

The amount of MDA significantly increased in the control and FF groups compared with the 0-hour group (*P* ≤ 0.01). However, MDA level was significantly (*P* ≤ 0.001) lower in the presence of FF than in the control (Table 3).

**Table 3:** Evaluation of lipid peroxidation (MDA) levels, total antioxidant capacity (TAC), and catalase activity in different groups of human spermatozoa. The data are presented as the mean ± SEM.

Groups*	MDA amount (nmol/sperm)	TAC amount (μmol/sperm)	Catalase activity (U/sperm)
0-hour	0.51 ± 0.01^a^	0.62 ± 0.01^a^	3.17 ± 0.01^a^
Control	1.19 ± 0.08^b^	0.39 ± 0.01^b^	1.72 ± 0.12^b^
FF (50%)	0.73 ± 0.10^c^	0.49 ± 0.01^c^	2.30 ± 0.16^c^

* 0-hour**:** sperm were evaluated before freezing; Control: sperm were evaluated after freezing; FF (50%)**:** sperm were frozen with FF (50%) and thawed after 2 weeks and evaluated.

Different subscripts (a, b, c) denote significant differences between groups (repeated measure analysis, *n* = 30 for each group, *P* < 0.05).

### Total antioxidant capacity (TAC)

Based on the results, the levels of TAC in the control and FF groups decreased significantly compared with the 0-hour group (*P* ≤ 0.01). However, in the group incubated with FF (50%), sperm TAC level was significantly (*P* ≤ 0.001) higher than the control group (Table 3).

### Catalase activity

Catalase activity decreased significantly (*P* ≤ 0.01) in the control and FF groups compared with the 0-hour group. However, in the group incubated with FF (50%), sperm catalase activity was significantly (*P* ≤ 0.001) higher than the control group (Table 3).

## Discussion

Our results showed that freezing–thawing induces negative effects on vital parameters of human spermatozoa compared with the 0-hour groups (before freezing), and follicular fluid could preserve sperm parameters and compensate for the adverse effects of freezing–thawing.

It has been reported that sperm freezing induces adverse effects on sperm and reduces its fertilizing ability. Consistent with our results, Oberoi *et al.* showed that human sperm motility decreased after freezing–thawing (Oberoi *et al.* 2014). Furthermore, it has been shown that the total and progressive motility, viability, and mitochondrial membrane potential significantly decrease in human normozoospermia samples and boar sperm after freezing and thawing (Zhang *et al.* 2021, Behmanesh *et al.* 2023).

The formation of ice crystals, dehydration, and osmotic pressure changes can be considered possible mechanisms of sperm damage during freezing–thawing (Blesbois *et al.* 2005). These factors can affect sperm membrane structure and mitochondrial function and disrupt biochemical processes involved in the production of ATP. Therefore, in addition to reducing plasma membrane and mitochondrial integrity, sperm motility and viability decrease (Kommisrud *et al.* 2020). In addition, cryoinjuries decrease the expression of some antiapoptotic proteins, such as BCL-2, and increase the expression of proapoptotic proteins, such as caspase-3 and BAX, resulting in decreased sperm viability (He *et al.* 2020).

On the other hand, cryopreservation-induced oxidative stress contributes to mitochondrial membrane peroxidation, decreases the MMP, disrupts ATP production, and consequently, impairs sperm motility and viability (Tiwari *et al.* 2022). During freezing–thawing, oxidative stress decreases sperm motility through direct effects on mitochondrial activity, disrupting intracellular enzymes and some axonemal proteins (Gualtieri *et al.* 2021). Lipid peroxides such as 4HNE (4-hydroxynonenal) and MDA (malondialdehyde), as a result of oxidative stress, can affect heat-shock proteins, axonemal proteins such as dyneins and tubulins (which are involved in sperm motility), and mitochondrial proteins, such as succinate dehydrogenase and ATP synthesis. Taken together, these events can have negative effects on the cytoskeleton, sperm motility, and viability (Peris-Frau *et al.* 2020).

In the present study, sperm freezing and thawing significantly decreased plasma membrane and acrosome integrity. During freezing, the formation of ice crystals inside and outside the cell induces cold shock and osmotic stress, which eventually damages the sperm plasma membrane and acrosome (Sieme *et al.* 2015). In addition, phase transitions of membrane lipids, which occur due to temperature changes, impair the function of membrane proteins, which are responsible for ion transport and metabolism. These changes reduce the function and integrity of the plasma membrane (Ozimic *et al.* 2023). Different enzymes, such as hyaluronidase enzyme (HYD) and sperm acrosome enzyme (ACE), play important roles in acrosome function. Freezing induces a series of changes in the plasma membrane and head ultrastructure, leading to a loss of these enzyme contents and a negative effect on the sperm acrosome (Sun *et al.* 2021).

High production of ROS during the freezing procedure induces membrane phospholipid peroxidation and rearranges the sperm glycocalyx, resulting in damage to sperm membrane structures, including lipids, proteins, and carbohydrates, and a decrease in plasma membrane integrity (Zhang *et al.* 2021). A decrease in membrane integrity disrupts its important role in cell signaling and leads to an imbalance in ions concentration inside cells. As a result, the levels of calcium can increase inside cells, resulting in the induction of premature acrosome reaction (Peris-Frau *et al.* 2020).

Furthermore, our results displayed that sperm freezing increases DNA fragmentation. Studies have shown that freezing–thawing increases the percentage of sperm DNA fragmentation and caspase-3 activity (Amor *et al.* 2018). Asa *et al.* reported that the percentage of DNA fragmentation increased after freezing (Asa *et al.* 2020). During the freezing and thawing process, intracellular ice crystal formation likely reduces nuclear DNA/protein interactions and leads to sperm DNA damage (Kopeika 2021). In addition, oxidative stress leads to the breaking of disulfide bonds in sperm protamine, which causes the loss of sperm nuclear protamine. Loss of sperm nuclear protamine increases the probability of DNA fragmentation and causes irreversible damage to sperm DNA (Flores *et al.* 2011). In addition, free radicals created during freezing can directly activate caspases and other apoptotic factors and increase DNA fragmentation and cell death (Karabulut *et al.* 2018).

It has been reported that cryopreservation changes the distribution or expression of proteins involved in ROS inhibition (Peris-Frau *et al.* 2020). On the other hand, high levels of polyunsaturated fatty acids (PUFAs) in the sperm membranes increase their susceptibility to ROS and peroxidative damage (Koppers *et al.* 2010).

Therefore, according to the results of the previous studies and also our research, it can be assumed that freezing–thawing likely induces oxidative stress and causes damage to sperm in freezing–thawing. The results of our study showed that the amount of MDA (a marker of lipid peroxidation) significantly increased and total antioxidant capacity and catalase activity significantly decreased after freezing and thawing compared with the 0-hour group (before freezing). Based on these results, therefore, oxidative stress may be one of the possible mechanisms of sperm damage during freezing–thawing in this study.

According to previous studies, supplementation of freezing medium with antioxidants and some cryoprotectants increases the efficiency of this method by reducing ROS-induced damage to sperm (Santonastaso *et al.* 2021). In this context, the results of the present study illustrated that the addition of 50% human FF to human semen before cryopreservation increased sperm motility, viability, plasma membrane integrity, TAC levels, and catalase activity and decreased the amount of MDA compared with the control group. Several studies in human and animal models have confirmed the positive effects of follicular fluid on spermatozoa. FF improves progressive motility and reduces the percentage of immotile sperm. In addition, FF may influence sperm capacitation, the final maturation step required for fertilization. Factors in FF, such as cholesterol acceptors and signaling molecules, may accelerate or modulate the capacitation process (Yao *et al.* 2000). FF can modulate the acrosome reaction. Optimal regulation of this reaction is essential for successful fertilization. In addition, components in FF, such as antioxidants, protect sperm from oxidative stress, thus improving sperm viability and lifespan. Higher viability translates to a greater chance of successful fertilization (Hasan *et al.* 2021). These antioxidants scavenge reactive oxygen species (ROS), which can damage sperm membranes and DNA, leading to reduced fertility. The protective effects of FF against oxidative stress may also improve sperm DNA integrity, leading to healthier embryos and improved pregnancy outcomes. Damage to sperm DNA can result in reduced fertilization rates, increased rates of miscarriage, and adverse effects on offspring health (Dorado-Silva *et al.* 2020).

Several factors that act as regulators of oxidative stress and antioxidant activity have been reported in FF (Singh *et al.* 2013). In addition, antioxidants such as peroxidase, superoxide dismutase, and glutathione are present in FF, which prevent lipid peroxidation and damage to the sperm plasma membrane (Fujimoto *et al.* 2011).

The results of the present study also revealed that the MMP and DNA integrity were greater in the group incubated with FF than in the control group. A likely mechanism by which FF can preserve sperm DNA integrity may be related to the presence of Bcl-2 (an antiapoptotic factor) in FF, which can prevent apoptosis and cell death. The Bcl-2 protein can also inhibit the release of cytochrome C and apoptosis-inducing factors from the mitochondrial membrane and thereby prevent apoptosis and DNA fragmentation (Park *et al.* 2014). FF increases the stability of sperm DNA by displacing normal protamine with a stable S–S disulfide bond, leading to a reduction in DNA denaturation (Condorelli *et al.* 2012, Keyser *et al.* 2021). While our results for the first time indicate a promising effect of FF on human sperm parameters during freezing–thawing, further research is necessary to fully evaluate its potential to improve the fertilizing ability of freeze–thawed sperm. Specifically, clinical trials are needed to determine optimal dosages, assess long-term outcomes, and ensure safety before the widespread implementation of assisted reproductive technologies.

## Conclusion

Our results showed that oxidative stress can be an important cause of sperm damage during freezing–thawing. In addition, the incubation of semen with FF before freezing can protect sperm against oxidative stress and also decrease sperm cryoinjury. Therefore, it can be concluded that antioxidant compounds and other appropriate factors in human follicular fluid likely suppress oxidative stress and thereby improve vital sperm parameters after thawing. However, further studies are necessary to determine the roles and detailed mechanisms of follicular fluid during freezing–thawing and its applicability in the clinic.

## Declaration of interest

The authors declare that there is no conflict of interest that could be perceived as prejudicing the impartiality of the work reported.

## Funding

This work did not receive any specific funding from public, commercial, or nonprofit funding centers.

## Author contribution statement

MM was responsible for the study design. MM, ESH, and MD-F contributed to sample collection and all experimental and statistical analyses, drafting the manuscript, and editing of the final manuscript.

## Data availability

The data are available from the corresponding author on reasonable request.
